# Local Anesthetic Ropivacaine Exhibits Therapeutic Effects in Cancers

**DOI:** 10.3389/fonc.2022.836882

**Published:** 2022-02-03

**Authors:** Peng Xu, Shaobo Zhang, Lili Tan, Lei Wang, Zhongwei Yang, Jinbao Li

**Affiliations:** ^1^Department of Anesthesiology, Shanghai General Hospital, Shanghai Jiao Tong University School of Medicine, Shanghai, China; ^2^Department of Anesthesiology, Gansu Provincial Maternity and Child Care Hospital, Lanzhou, China

**Keywords:** drug repurposing, local anesthetics, ropivacaine, anticancer, mechanisms

## Abstract

Despite the significant progress in cancer treatment, new anticancer therapeutics drugs with new structures and/or mechanisms are still in urgent need to tackle many key challenges. Drug repurposing is a feasible strategy in discovering new drugs among the approved drugs by defining new indications. Recently, ropivacaine, a local anesthetic that has been applied in clinical practice for several decades, has been found to possess inhibitory activity and sensitizing effects when combined with conventional chemotherapeutics toward cancer cells. While its full applications and the exact targets remain to be revealed, it has been indicated that its anticancer potency was mediated by multiple mechanisms, such as modulating sodium channel, inducing mitochondria-associated apoptosis, cell cycle arrest, inhibiting autophagy, and/or regulating other key players in cancer cells, which can be termed as multi-targets/functions that require more in-depth studies. In this review, we attempted to summarize the research past decade of using ropivacaine in suppressing cancer growth and sensitizing anticancer drugs both *in-vitro* and *in-vivo*, and tried to interpret the underlying action modes. The information gained in these findings may inspire multidisciplinary efforts to develop/discover more novel anticancer agents *via* drug repurposing.

## Introduction

**Figure 1:**
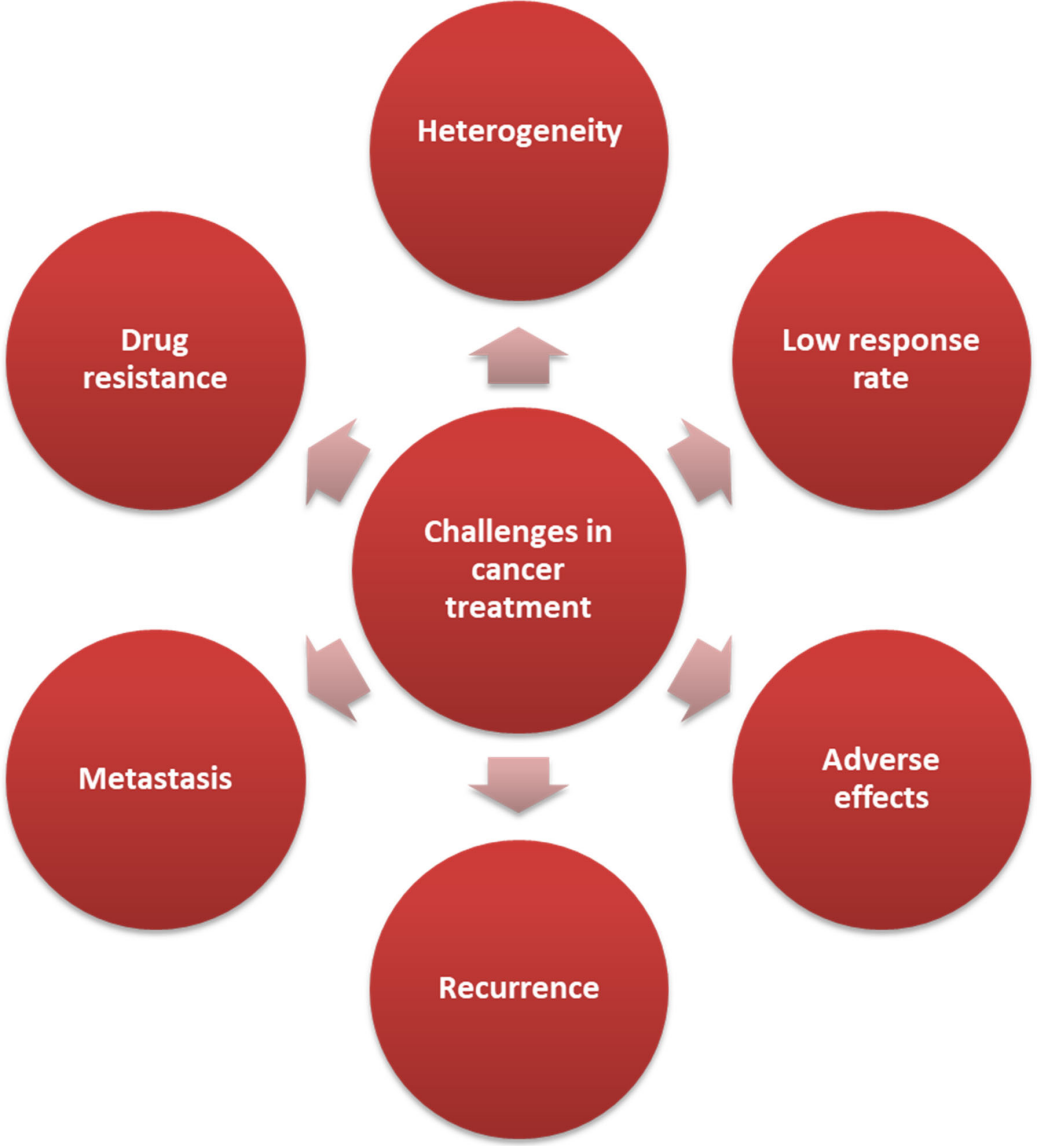
The effective cancer treatment can be undermined by many key challenges.

Cancer has become a global health burden in both developing and developed countries. Despite the significant progress of chemotherapies and immunotherapies, unexpected low response rate, unfavorable adverse effects, multidrug resistance (MDR) and cancer recurrence are among the major challenges that undermine effective cancer treatment as summarized in **Figure 1** (1–4). New drugs with novel structures and/or mechanisms and novel therapeutic strategies remain unmet clinical needs to tackle these issues. The discovery and development of one new drug, especially *de novo* drug discovery, may approximately take at least ten years and one billion dollars, rendering it a highly challenging and risky task due to the fact of high attrition rates (5–7). Potential strategies that may serve as shortcuts in drug discovery are 1) old drugs repurposing, 2) co-crystallization between lead compound and its target protein, which may fasten the identification and optimization of drug candidates, 3) artificial intelligence (AI) and machine learning, and 4) others such as high throughput screening (HTS) in natural products or other commercial available compound libraries (8–12). Recently, old drug repurposing has been proved to be a feasible strategy to develop new drugs from those approved drugs by defining new indications (13–18). More importantly, these approved drugs have already been evaluated in humans to possess favorable profiles of pharmacokinetic, pharmacodynamic profiles and safety, as well as controllable/acceptable adverse/toxic effects, which is a procedure of time-consuming (19). Drug repurposing may indeed shorten the overall time in developing and launching a new drug, and may substantially relief the financial burden as compared to *de novo* drug development (16, 17, 20, 21).

Retrospective clinical studies have suggested that the application of local anesthetics can improve the treatment outcomes of certain cancer patients in pain control and, more strikingly, in suppressing cancer growth (22, 23). In the past decade, researchers have studies intensively in discovering new agents with anticancer activities among local anesthetics, finding that several anesthetics possess broad-spectrum anticancer potencies (24), such as lidocaine (25–27), procaine (28–30), ropivacaine (31–35) and its stereoisomers bupivacaine (36, 37) and levobupivacaine (38, 39). Following our previous review of lidocaine in cancer treatment (11), in the current review, we aimed to summarize the anticancer studies of ropivacaine, another amide-linked local anesthetic (same as lidocaine) that has been widely used in perioperative period as a long acting local anesthetic (40, 41). Structurally, ropivacaine is the S-enantiomer of bupivacaine, while it has a weaker cardiotoxicity and other toxic effects than bupivacaine when used as an anesthetic (42, 43). Ropivacaine, when repurposed as anticancer agent that is administered by single or combination, inhibits cancer cells growth, proliferation, invasion and migration through multiple mechanisms, showing great potential in cancer treatment.

## Ropivacaine Demonstrates Anticancer Efficacies

Growing evidence has suggested that at certain concentrations/doses, ropivacaine can work as an anticancer agent by monotherapy or combination. In this section, we summarized these studies categorized by the functions such as inhibiting proliferation or invasion, etc. Compared to lidocaine that has been evaluated in various cancer types *in-vitro* and *in-vivo* (11), relatively fewer studies were conducted using ropivacaine in cancer treatment. By far, it’s still not clear about the direct interactions of local anesthetics on cancer cells. Further applications can be significantly expanded after deciphering the exact mechanism and the target of ropivacaine.

## Ropivacaine Inhibits Cancer Cells Proliferation

### *Via* Regulating Ras and RhoA Signaling Pathway

There are studies that indicated the anticancer effects of ropivacaine are independent of its role in regulating sodium channel (44). RhoA and Ras are two important members of Ras superfamily that regulates many aspects of cancer cell biology including cell division, proliferation and migration, whose inhibitors hold promising activity against cancer (45, 46). Zheng et al. (44) found that ropivacaine (0.25, 0. 5, 1 and 2 mM) inhibited the proliferation and migration of human melanoma A375 and A431 cells in a concentration-dependent manner *via* inducing apoptosis (44). More importantly, it (0.5 mM) could also serve as chemosensitizer as it markedly enhanced the potencies of vemurafenib and dacarbazine, two widely used drugs in melanoma treatment, suggesting a broader screening of its potential in drug resistant cancers. Interestingly, its isomer bupivacaine didn’t show such sensitizing effect as ropivacaine. These effects were independent of sodium channel but were mediated by the inhibition of RhoA and Ras, which can be reversed by pre-treatment with the activator of Ras and Rho, calpeptin (44). The Western blot analysis also showed that ropivacaine treatment (1 and 2 mM) not only caused the down-regulation but also inhibited the activities of several downstream signaling of Ras and RhoA, such as MAPK/ERK Kinase (MEK) and myosin phosphatase target subunit 1 (MYPT1) MLC, further verifying its mechanism (44).

### *Via* Regulating Integrin Alpha-2 (ITGα2) and ITGβ1

ITGα2 is a key protein that closely participates in cell adhesion. Serving as a therapeutic target, ITGA2 is found to be overexpressed in certain cancer cell lines and tumor tissues, which may cause the promotion of cancer aggression (47, 48). Ropivacaine (2.5-40 μM) inhibited the proliferation of gastric cancer AGS and BGC-823 cells as shown in a study by Qin et al. (49). Mechanistic study indicated that ropivacaine inhibited the expression of ITGA2 in a concentration-dependent manner, resulting in significant apoptosis as supported by the down-regulated anti-apoptotic B-cell lymphoma 2 (Bcl-2), and up-regulated pro-apoptotic Bcl-2-associated X protein (Bax), cleaved caspase 3/9. Importantly, these effects could be reversed by the overexpression of ITGα2, indicating that ropivacaine’s anticancer effects were mediated by inhibiting ITGα2 (49). In this study, ropivacaine was also found to suppress the invasion and metastasis of human papillary thyroid cancer (PTC) TPC-1 cells, suggesting it a multifunctional agent (49).

Another recent study by Wang and Li (50) showed that ropivacaine (200, 400, 800 μM) induced apoptosis and inhibited the proliferation and migration of colon cancer HCT116 and SW620 cells by targeting another subunit of integrin, ITGβ1 (50).

### *Via* Regulating Wnt/β-Catenin

Cancer stem cells (CSCs) are a subpopulation of cancer cells that have self-renewing and highly proliferative properties, which can often cause cancer recurrence and drug resistance (51–53). Wnt/β-catenin pathway plays critical role in regulating the pluripotency and renewal of CSCs. In addition, Wnt/β-catenin is found to be dys-regulated in cancer patients, indicating its potential therapeutic implication (54, 55). At clinical relevant concentrations, ropivacaine exhibits inhibitory effects towards CSCs. A study conducted by Li et al. (28) showed that ropivacaine (10, 50 and 100 μM) inhibited leukemia stem cell (LSC) stronger than normal hematopoietic stem cell (HSC), although ropivacaine was found to be less potent than lidocaine or bupivacaine (56). At the same concentrations, ropivacaine significantly repressed the colony numbers as well as the serial replating of LSC, likely *via* inhibiting Wnt/β-catenin as confirmed by Western blot analysis, suggesting its potential capabilities in inhibiting CSCs and warranting further studies (56).

### *Via* Regulating Autophagy Through Vascular Endothelial Growth Factor (VEGF)-A and Signal Transducer and Activator of Transcription 3 (STAT3)

Autophagy is a biological procedure that prompts cancer cells to respond nutrition changes by degrading and then recycling the intracellular biomacromolecules, serving as a promising therapeutic target in cancer (57–59). Combinational therapy of ropivacaine has also been attempted to inhibit pain relief and tumor growth simultaneously. Zhang et al. (20) developed a formulation of liposomes composed with ropivacaine (named as Rop-DPRL), and these liposomes, when combined with nutrition deprivation which may lead to activated autophagy, can suppress the tumor growth of melanoma B16 cells xenograft model and relieve the cancer pain (60). Further study indicated that these effects were mediated by reducing the expression of VEGF-A, and inhibiting the phosphorylation of STAT3 (60).

### *Via* Apoptosis-Associated Pathways and Cell Cycle Arrest

Most of cancer cells die due to apoptosis induced by different therapeutic strategies. Apoptosis, the programmed cell death, can be categorized into external and internal apoptosis which are initiated *via* distinct pathways. Both of them can serve as therapeutic targets that can be attacked by small-molecule drugs or macromolecule drugs *via* intervening the key components, e.g., either activating the pro-apoptotic proteins or suppressing the anti-apoptotic ones (61, 62).

One of the hallmarks of cancer cells is the uncontrollable cell division and proliferation. Key enzymes such as cyclin-dependent kinases (CDKs) and members of cyclins are dynamically stimulated to regulate the active cell cycle, rending them to be attracting and druggable targets (63, 64). Growing evidence has showed that ropivacaine can kill cancer cells *via* inducing apoptosis or cell cycle arrest.

Castelli et al. (65) evaluated the cytotoxicity of ropivacaine on drug-resistant human triple-negative breast cancer MDA-MB-231 cells, and melanoma A375 cells (65). Ropivacaine (5-1000 µM) was found to concentration-dependently inhibit the proliferation of both cell lines, and suppressed the migration as confirmed by the transwell assay. Ropivacaine induced significant apoptosis by up-regulating the cleaved caspase 3 and 9, and it also caused cell arrest *via* inhibiting the expression of cyclins B2, D1 and E. These effects suggested that ropivacaine suppressed cancer cells proliferation *via* cell cycle arrest and activating apoptosis pathway (65).

Another study showed that ropivacaine possessed similar activity in human non-small cell lung cancer (NSCLC) A549 and H520 cells (66). Ropivacaine (2-12 mM) inhibited the cell viability, suppressed the invasion and migration at 4.06 and 2.62 mM (ED_50_ values) *via* inducing G0/G1 phase arrest and apoptosis by down-regulating anti-apoptotic but up-regulating pro-apoptotic proteins, provoking DNA damage and reactive oxygen species (ROS) production through activating mitogen-activated protein kinase (MAPK) pathways (66). It’s worth noting that this study using much higher concentration of ropivacaine that that of in Castelli et al.’s study (65), which is a common issue using local anesthetic as anticancer agent, further pharmacokinetic studies are needed.

Li et al. (28) reported that at plasma concentrations (10, 35 µM, much lower than 1 mM) for 72 h, ropivacaine failed to decrease cell viability and migration of breast cancer MDA-MB-231 and MCF7 cells, while at higher concentrations (more than 1 mM), it significantly inhibited cell viability and showed cytotoxicity without affecting the viability of a non-cancerous breast cell line, MCF10A, suggesting its selective profile (67). At 10-fold plasma concentrations, ropivacaine suppressed the migration of MDA-MB-231 by inducing cell arrest at the S phase (64).

Another similar result was found that ropivacaine at 1 mM decreased the viability and proliferation of hepatocellular carcinoma (HCC) HuH7 cells while spared the well-differentiated HepaRG cells (68). The levels of mRNA of several key cell-cycle regulators, including cyclin A2, B1/2, and CDK1, as well as the marker of proliferation Ki-67 (MKI67) were significantly suppressed, indicating a cell arrest-mediated mechanism (68).

Mitochondria are pivotal organelles in cancer cells for their roles in ATP production (mitochondrial respiration) and endogenous apoptosis pathway initiating and regulating, rendering them to be attractive therapeutic targets (67, 69, 70). Several studies have indicated that ropivacaine could exert its anticancer effects through inducing mitochondria-mediated apoptosis and/or impacting the respiration pathway (71, 72). In HCC Bel7402 and HLE cells, in the concentration- and time-dependent manners, ropivacaine markedly suppressed the cells proliferation and migration *via* damaging mitochondria by inducing endogenous apoptosis event as confirmed by the up-regulated caspase 3/9, apoptotic protease activating factor-1 (Apaf-1) and released cytochrome C from mitochondria and down-regulated anti-apoptotic Bcl-2 (73). Another study conducted by Yang et al. (32) showed that at clinically relevant concentrations, ropivacaine was able to suppress the angiogenesis of human lung tumor-associated endothelial cells (HLT-EC) *via* disturbing the complex II located in the mitochondrial respiration chain. The damaged mitochondrial respiration caused by ropivacaine further leads to ATP depletion, overproduced ROS and finally lethal damages to cells (74).

Another study by Gong et al. (33) showed that ropivacaine (0.5 and 1 mM) inhibited the activities of complex I and II in mitochondrial respiration chain in breast cancer MDA-MB-468 and SkBr cells, leading to the repressed growth, survival and colony formation through inducing oxidative stress (75). Further study indicated that ropivacaine could work as a chemo-sensitizing agent since it (0.5 mM) can enhance the sensitivity of 5-fluorouracil (5-FU) *via* inhibiting the phosphorylation of Akt, mammalian target of rapamycin (mTOR) and ErbB3 receptor-binding protein 1 (EBP1) (75).

Ropivacaine also exerts inhibitory effects towards mesenchymal stem cells (MSCs) which possess self-renewing property that may contribute in wounds healing and tumor growth (71, 72, 76). At 100 μM, ropivacaine induced proliferation inhibition, cell arrest at the G0/1-S phase, resulting in less colony formation and delayed wound healing *via* impacting mitochondrial respiration and reducing ATP production (77).

### *Via* Regulating Extracellular Signal-Regulated Kinases 1/2 (ERK1/2)

ERK1/2 signal pathway is one of the central players in regulating cell biology, such as proliferation, differentiation, autophagy, stress response apoptosis and survival (78). Several selective ERK1/2 inhibitors are undergoing clinical trials, showing their great potentials in certain cancers treatment (79). Yang et al. (80) found that ropivacaine (1 mM) significantly inhibited the proliferation and migration of gastric cancer AGS and HG-27 cells *via* down-regulating phosphorylated ERK1/2 (80). Further studies are necessary to elucidate the details of impacted signal pathway and associated cancer cell biology, e.g., the interaction of ERK1/2 down-regulation with autophagy (81), apoptosis (82), and cell cycle (83), etc.

### *Via* Micro RNAs/Long Non-Coding RNAs (lncRNAs) and Associated Signaling Pathways

Micro RNAs have drawn profound attentions for their roles in regulating cancer progression and migration, serving as therapeutic target (84). Zhang et al. (20) found that ropivacaine could up-regulate miR-520a-3p that can further suppressed the expression of WEE1 and phosphorylated PI3K, leading to concentration- and time-dependent inhibition of the proliferation of gastric cancer AGS and BGC-823 cells, suppression of the migration and invasion (85). More importantly, in the AGS cells xenograft mouse model, ropivacaine (20, 40, and 60 μM/kg) significantly reduced tumor growth, accompanied with up-regulated miR-520a-3p and decreased WEE1 and phosphorylated PI3K (85).

In breast cancer MDA-MB-231 and MCF-7 cells, ropivacaine (1 mM) induced apoptosis, leading to the time-dependent inhibition of the proliferation, and the reduction of the colony formation, as well as decreased cell invasion and migration (86). Ropivacaine was confirmed to up-regulate miR-27b-3p and its target gene *YAP* to exert its anticancer effects. Ropivacine (40 μM/Kg) inhibited the tumor growth of MDA-MB-231 cells xenograft model, which can be reversed by co-treatment of miR-27b-3p inhibitor (86).

Recently, lncRNAs have shown great potentials as key players in gene regulation and cancer progression. MEG2 lncRNA regulates epigenetic modifications through interacting with chromatin-modifying complexes, acting as a tumor suppressor that is down-regulated in various types of cancer (87). As a central player in cell biology and a therapeutic target, STAT3 is a transcription factor that regulates cell differentiation, proliferation and apoptosis, resulting in promoting cancer progression (88). Chen et al. (89) reported that ropivacaine (0.25, 0.5 and 1 mM) possessed inhibitory effects to cervical cancer SiHa, Caski cells *via* suppressing the expression of cyclin D1 and survivin, an anti-apoptotic protein (90, 91), by abrogating the phosphorylation and transcriptional activation of STAT3 whose overexpression could reverse the cytotoxicity of ropivacaine (89). These effects were mediated by up-regulating MEG2 and down-regulating microRNA96, suggesting ropivacaine as a potential therapeutic agent for cervical cancer (89).

## Ropivacaine Inhibits Cancer Cells Invasion And Migration

As discussed above, ropivacaine at certain concentrations/doses could not only suppress the proliferation, but also the invasion and migration *via* similar multiple mechanisms. While there are also studies indicated that ropivacaine can only inhibit the invasion and migration, but not be able to kill cancer cells, probably due to the applied different concentrations, e.g., lower concentrations.

### *Via* Regulating Sodium Channels

Proteins in regulating sodium channel such as NaV1.5 voltage-gated Na^+^ channel (VGSC), can also prompt the tumorigenesis including the proliferation and metastasis of cancers (92, 93). Certain types of metastatic cancer cells, including breast and colon cancer cells, express high level of NaV1.5 VGSC, which may lead to poor prognosis of patients (93–96). Consequently, the block of NaV1.5 VGSC leads to the cease of cancer cell invasion (93, 97). As a local anesthesia, ropivacaine works by inhibiting sodium channel that mediates the pain signal transduction (98). Accordingly, ropivacaine can also well exert its anticancer effects *via* the same mechanism of action through the inhibition of NaV1.5 VGSC. A study conducted by Baptista-Hon et al. (97) found that, ropivacaine blocked the NaV1.5 VGSC of both neonatal and adult splice variants in colon cancer SW620 cells, with IC_50_ values of 2.5 and 3.9 μM, respectively. Consequently, ropivacaine inhibited the invasion of SW620 cells (IC_50_ of 3.8 μM), suggesting its potential application in controlling colon cancer invasion (97).

In 2015, a systematic review by Koltai et al. was published, aiming in searching regulators of VGSCs (NaV1.1 to Nav1.9) and the potentials of their regulators in suppressing invasion and metastasis of cancers (99). In this review, they reported that a couple of local anesthetics, such as ropivacaine and lidocaine, as well as many other drugs, may serve as anticancer agents in suppressing metastasis and invasion of cancer cells (99). However, their further applications in clinic remain to be unveiled (99). This review also suggests a wider screening of this type of approved drugs for their potential in cancer treatment.

### *Via* Attenuating the Axis of Rac1/c-Jun N-Terminal Kinase (JNK)/Paxillin/Focal Adhesion Kinase (FAK)

A study in Zheng’s group (2018) showed that at lower concentrations (less than 200 μM), ropivacaine didn’t impact cancer cell growth and survival but suppress the cell migration (100). As shown in this research, at the clinically relevant concentration (50, 100 and 200μM), ropivacaine inhibited the migration of esophageal cancer OE33, ESO26 and FLO-1 cells *via* decreasing the activities of GTPases of RhoA, Rac1 and Ras, and inhibiting the prenylation, which were independent of sodium channel. This work demonstrated that the potent anti-migratory effect of ropivacaine in esophageal cancer was mediated by attenuating the axis of Rac1/JNK/paxillin/FAK and prenylation-dependent migratory signaling pathways (100).

### *Via* Regulating Matrix Metalloproteinase 9 (MMP9)/Akt/FAK

MMP9 is a key protein that plays key role in cancer cells invasion, which serves as a prognostic biomarker in certain cancer patients, implying its potential role as a therapeutic target (101–103). In NCI-H838 lung adenocarcinoma cells, ropivacaine (1 nM-100 μM) significantly reduced TNFα-induced activation/phosphorylation of Akt, FAK, caveolin-1 as well as MMP9 *via* attenuating tyrosine protein kinase Src-dependent inflammatory pathway. Ropivacaine (1 μM) completely inhibited the invasion of NCI-H838 cells, suggesting its potential in suppressing the metastasis (104).

Similar results were also confirmed by Piegeler et al. (2012) that ropivacaine, through its anti-inflammatory effects, suppressed the Src and vascular intercellular adhesion molecule-1 (VCAM-1), two important key players in tumor growth and metastasis (104, 105). Ropivacaine (100 μM for 20 min) decreased the Src activity by 62% through decreasing Src-activation and intercellular adhesion molecule-1 phosphorylation (106).

### *Via* Nuclear Factor Kappa-Light-Chain-Enhancer of Activated B Cells (NF-κB) Pathway

NF-κB pathway regulates cancer cell proliferation, survival, and angiogenesis, playing pivotal role in cell biogenic activities and serving as an attracting target in cancer treatment (105). Su et al. (107) found that ropivacaine (10 μM), when combined with tumor necrosis factor α (TNFα), caused the inhibition of adhesion of three cancer cell lines, human hepatoma HepG2 cells, human colon cancer HT-29 cells and human leukemic monocyte THP-1 cells. These effects were mediated by down-regulating the expression of CD62E, a key protein in regulating adhesion (106, 108). Further mechanistic study showed that ropivacaine significantly suppressed the expression of several key components of NF-κB pathway, including the phosphorylation of p65, IκBα and IKKα/β, indicating that ropivacaine decreased the adhesion of cancer cells *via* modulating CD62E expression by inhibiting the NF-κB pathway (107).

### *Via* DNA Demethylation

DNA methylation is a procedure through which bases are modified by methyl group, which is found to be highly active in many cancers (109). The inhibitors of DNA methylation can produce anticancer potencies and several of these inhibitors have been approved by FDA (110). Ropivacaine showed epigenetic regulatory effects *via* modulating DNA methylation. As shown in Lirk et al.’s study (111), ropivacaine at clinically relevant concentrations (3 and 30 µM) didn’t directly kill but decrease the DNA methylation in breast cancer BT-20 cells which lead to lower tumorigenesis properties (111).

## Clinical Studies

A recent retrospective cohort study of 215 pancreatic cancer patients by Chen et al. (112) showed that 0.375%-0.5% of intraoperative epidural ropivacaine significantly improve the overall survival (112). Several clinical trials have already been conducted by applying ropivacaine as an adjuvant therapy in shortening the recovery time and other beneficial effects in surgical cancer patients. A clinical study revealed recently (2020) that in liver cancer patients, ropivacaine, when combined with dezocine, could significantly shorten the recovery time of anesthesia and inhibit pain factors secretion, with markedly less adverse reaction, and this combination therapy could reduce stress response, promote patients’ postoperative recovery after cancer surgery (113). Another study (NCT02256228) showed that *via* the anti-inflammatory and analgesic effects, intraperitoneal ropivacaine in ovarian cancer patients could prompt the postoperative recovery and shorten the time for chemotherapy, which may lead to better overall recovery (114). Similar results were also observed in breast cancer patients who underwent surgery. This study (NCT02691195) showed that in the treatment group, 25 ml of 0.5% ropivacaine could improve the quality of recovery as confirmed by analyzing the 40-item questionnaire, leading to higher patient satisfaction (115). Wang et al. (31) reported that ropivacaine treatment might improve the postoperative cognitive dysfunction in patients following thoracotomy for esophageal cancer by down-regulating inflammatory cytokines such as IL-6 and TNFα (116).

Recently (2020), a study conducted in New Zealand to explore the long term of application of intraperitoneal ropivacaine in colonic cancer patients has been reported. This study had enrolled 60 patients of both benign and malignant colon cancer (stages I-III), and was analyzed by evaluating the overall survival, disease-free survival and recurrence. However, the revealed results showed that the treatment group by ropivacaine didn’t exhibit better overall survival or reduced mortality than the placebo group treated by 0.9% saline solution. And even worse, higher incidence of cancer-specific mortality was found in the ropivacaine-treated group, indicating no beneficial effects by applying intraperitoneal ropivacaine in patients with colonic malignancy (117). While further studies are clearly needed to explore the indications, e.g., earlier stages of cancers, and certain combinational therapy with ropivacaine.

## Discussion And Future Perspectives

**Figure 2:**
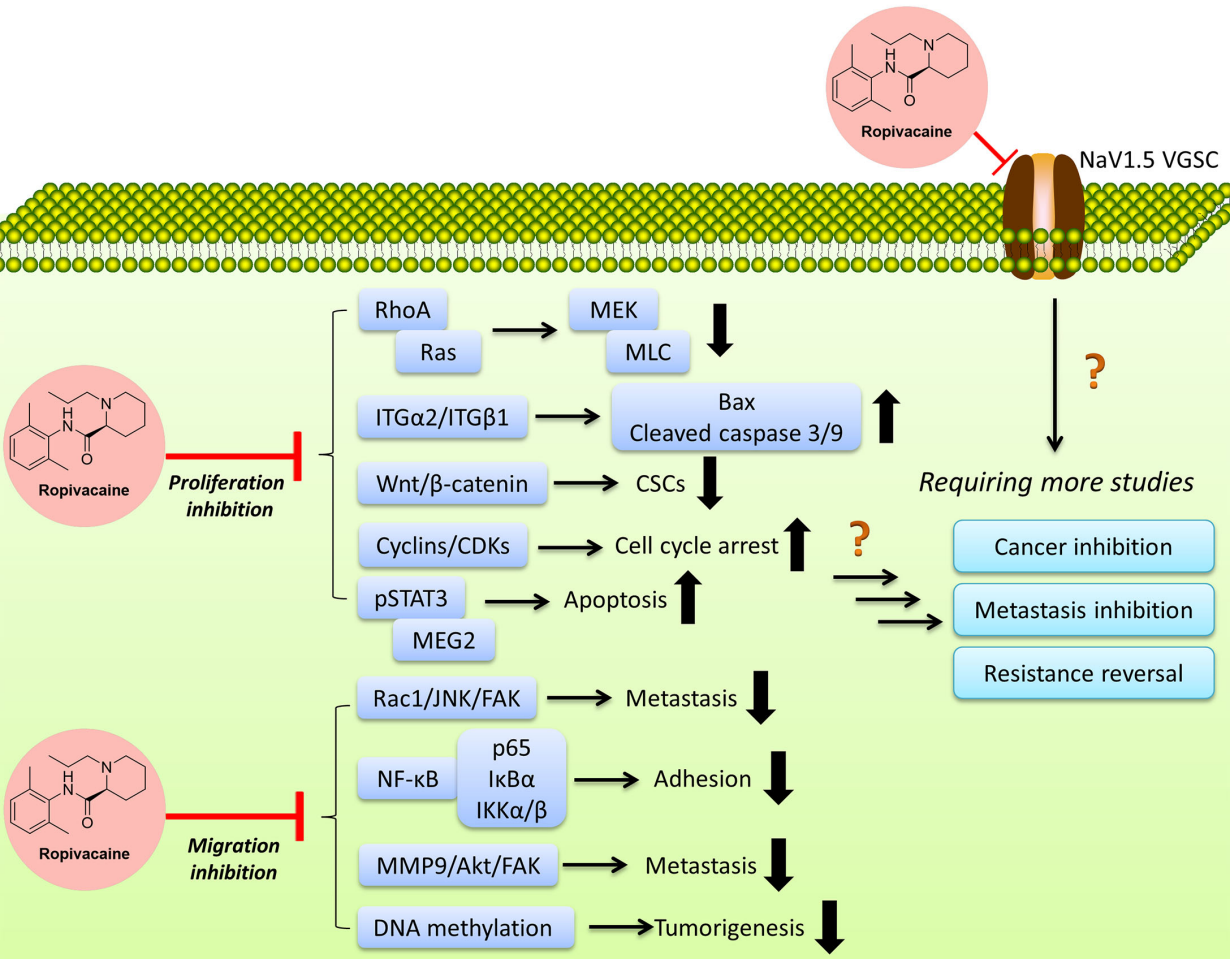
Ropivacaine suppresses cancer cells *via* multiple targets/functions by modulating multiple signal pathways. More efforts are needed to reveal the full map of the mechanisms.

The above studies indicate that local anesthetic ropivacaine may benefit cancer patients by two ways, 1) inhibit the proliferation or suppresses the migration of cancer cells as we summarized in **Table 1** and **Figure 2**, and 2) shortens the recovery times and improve the quality of life.

**Table 1:** Summary of ropivacaine in cancer treatment.

Targets/Mechanisms	Efficacies	Refs
Ras superfamily	Sensitizing vemurafenib and dacarbazine	(44)
ITGα2 and ITGβ1	Inhibiting the proliferation of AGS, and BGC-823 cells Inhibiting proliferation and migration of HCT116 and SW620 cells	(49, 50)
CSCs/Wnt/β-catenin	Inhibiting the proliferation of LSC	(56)
Autophagy/VEGF-A/STAT3	Inhibiting B16 cells xenograft tumor growth	(60)
Apoptosis-associated pathways	Inhibiting the proliferation and migration of MDA-MB-231 and A375 cells Inhibiting the invasion and migration of A549 and H520 cells Inhibiting the proliferation and migration of Bel7402 and HLE cells Inhibiting the capillary formation and growth of HLT-EC Inhibiting the proliferation of MDA-MB-468 and SkBr cells and sensitizing 5-FU	(65, 66, 73–75)
Cell arrest	Inhibiting the proliferation and migration of MDA-MB-231 and MCF7 cells Inhibiting the proliferation of HuH7 cells	(67, 68)
ERK1/2	Inhibiting the proliferation and migration of AGS and HG-27 cells	(80)
miR-520a-3p	Inhibiting the proliferation of gastric cancer AGS and BGC-823 cells	(84)
miR-27b-3p	Inhibiting MDA-MB-231 cells *in-vitro* and *in-vivo*	(85)
miR96/MEG2/pSTAT3	Inhibiting the proliferation of SiHa, Caski cells	(89)
Sodium channel	Inhibiting the invasion of SW620 cells	(97, 99)
Rac1/JNK/paxillin/FAK	Inhibiting the migration of OE33, ESO26 and FLO-1 cells	(95)
NF-κB	Inhibiting the adhesion of HUEVC	(107)
MMP-9/Akt/FAK	Inhibiting the invasion of NCI-H838 cells	(104)
DNA demethylating	Suppressing tumorigenesis properties	(111)

The studies performed *in-vitro* and *in-vivo* have proved that ropivacaine represses the cancer cells invasion and migration at lower concentrations (usually less than 200 μM) (100), while it suppresses cancer growth or kills cancer cells *via* various acting modes at higher concentrations/doses (mostly more than 1 mM) (118). However, the concentrations/doses vary when it comes to different cancer cells, requiring further in-depth studies for a clear therapeutic window in certain cancer type. In addition, ropivacaine appears to be an enhancer of the sensitivity of certain chemotherapy, suggesting its potential in the treatment of certain resistant cancers (44, 75). The information above may 1) suggest an appealing strategy in screening and identifying certain combinational therapy with ropivacaine, and 2) evoke a broad screening among local anesthetics and related drugs for cancer treatment. However, we cannot overstate the therapeutic implication until more results especially *in-vivo* and clinical studies are revealed.

In addition to its role in killing cancer cells, ropivacaine also improves the quality of life of cancer patients who have undergone surgical treatment. Many retrospective studies conducted among cancer patients upon treatment of local anesthetics including lidocaine and ropivacaine demonstrate a favorable trend of decrease in tumor metastasis and recurrence. While the clinical trials focusing on the anticancer effects of ropivacaine have yielded limited successes, there are still several ongoing trials for cancer-related diseases (see at http://6tt.co/tjEU on ClinicalTrials.gov), its potentials in cancer treatment remain to be fully revealed.

One interesting finding is the difference of anticancer efficacies of the analogs or isomers of ropivacaine. Ropivacaine and bupivacaine are optical isomers, while they possess different potentials and targets, e.g., GTPases (42), though they also exhibit similar effects in regulating certain targets such as hypoxia-inducible factor 2α (HIF-2α) signaling (115). More efforts are needed to decipher the underlying mechanisms, such as the binding targets, the network through which ropivacaine regulates certain signals transduction in cancers, etc. The application of ropivacaine in cancer treatment is still in its infant stage, more effects are clearly needed to define its indications and administration strategies (either single use or combination). Meanwhile, there are still many questions to be answered, such as the exact targets, and the acting concentrations/doses, more efforts (such as more *in-vivo* models, combinations, and certain rescue experiments) are needed to fill the blanks to obtain a full view (**Figure 2**). As per the reported studies, ropivacaine appears to be a multi-target or multi-functional compound. Under clinical relevant (achievable) concentrations/doses, it exerts anti-metastatic, anti-CSCs *via* regulating sodium channel, anti-inflammatory function and signaling pathways that regulate these two and other associated pathways, leading to the inhibition of signaling transduction which may contribute in the metastasis and maintenance of CSCs (94). Under multiple-fold of clinical-achievable concentrations/doses, ropivacaine is able to kill cancer cells by suppressing key players (proteins or signal pathways) in prompting cancer cells growth, proliferation and migration, including those key proteins in regulating cell cycle, apoptosis, mRNA, epigenetics, autophagy, etc. (64, 116).

It’s noteworthy that ropivacaine appears to exert its anticancer effects *via* the regulation of ITGα2 and/or members of Ras superfamily, because in the original studies, two pivotal experiments, such as the overexpression of ITGα2 (49) or the co-treatment of calpeptin, an activator of Ras and Rho (44), although other pathways can’t be excluded. Another interesting thing is among all affected pathways, several of them are membrane-associated proteins, such as NaV1.5 VGSC, as well as ITG members, indicating that ropivacaine might preferably attack membrane proteins which require further investigations. Again, future studies of the direct interaction between associated proteins and ropivacaine are warranted, which may help finally explain and identify its exact targets/mechanisms.

## Conclusions

Ropivacaine exerts anticancer and chemotherapeutic re-sensitizing effects, showing potentials in benefiting certain cancer patients. Further studies are warranted to explore the mechanisms, combinations and indications.

## Author Contributions

PX conceived the idea. PX, SZ, LT, and LW wrote the manuscript. PX, SZ, and JL revised the manuscript. All authors read and approved the final manuscript.

## Conflict of Interest

The authors declare that the research was conducted in the absence of any commercial or financial relationships that could be construed as a potential conflict of interest.

## Publisher’s Note

All claims expressed in this article are solely those of the authors and do not necessarily represent those of their affiliated organizations, or those of the publisher, the editors and the reviewers. Any product that may be evaluated in this article, or claim that may be made by its manufacturer, is not guaranteed or endorsed by the publisher.
